# 5-Chloro-4′-ethyl-3*H*-spiro­[1,3-benzo­thia­zole-2,1′-cyclo­hexa­ne]

**DOI:** 10.1107/S1600536812016479

**Published:** 2012-04-21

**Authors:** Mehmet Akkurt, Gökçe Cihan-Üstündağ, Gültaze Çapan, Sevim Türktekin-Çelikesir, Muhammad Nawaz Tahir

**Affiliations:** a Department of Physics, Faculty of Sciences, Erciyes University, 38039 Kayseri, Turkey; b Department of Pharmaceutical Chemistry, Faculty of Pharmacy, Istanbul University, 34116 Beyazıt, Istanbul, Turkey; c Department of Physics, University of Sargodha, Sargodha, Pakistan

## Abstract

In the title compound, C_14_H_18_ClNS, the 2,3-dihydro-1,3-thia­zole ring adopts an envelope with the S,N-bound C atom at the flap and the cyclo­hexane ring adopts a chair conformation. In the crystal, N—H⋯S hydrogen bonds with *C*(5) motifs connect the mol­ecules into chains parallel to the *c* axis.

## Related literature

For the pharmacological activity of benzothia­zole derivatives, see: Coudert *et al.* (1988 ▶); Karalı *et al.* (2010 ▶); Palmer *et al.* (1971 ▶). For standard bond lengths, see: Allen *et al.* (1987 ▶). For the graph-set analysis of hydrogen bonding, see: Bernstein *et al.* (1995 ▶). For ring-puckering analysis, see: Cremer & Pople (1975 ▶).
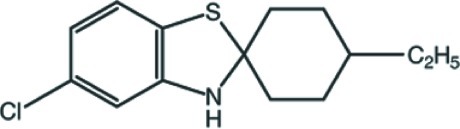


## Experimental

### 

#### Crystal data

C_14_H_18_ClNS
*M* *_r_* = 267.81
Orthorhombic, 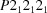

*a* = 8.989 (3) Å
*b* = 11.163 (4) Å
*c* = 13.722 (4) Å
*V* = 1376.9 (8) Å^3^
*Z* = 4
Mo *K*α radiation
μ = 0.41 mm^−1^
*T* = 296 K
0.35 × 0.28 × 0.25 mm

#### Data collection

Bruker Kappa APEXII CCD diffractometer
Absorption correction: multi-scan (*SADABS*; Bruker, 2005 ▶) *T*_min_ = 0.872, *T*_max_ = 0.903
7077 measured reflections
3203 independent reflections
2724 reflections with *I* > 2σ(*I*)
*R*_int_ = 0.020

#### Refinement

*R*[*F* ^2^ > 2σ(*F* ^2^)] = 0.038
*wR*(*F* ^2^) = 0.092
*S* = 1.03
3203 reflections
159 parameters
1 restraint
H atoms treated by a mixture of independent and constrained refinement
Δρ_max_ = 0.32 e Å^−3^
Δρ_min_ = −0.35 e Å^−3^
Absolute structure: Flack (1983 ▶), 1320 Freidel pairs
Flack parameter: 0.02 (8)

### 

Data collection: *APEX2* (Bruker, 2009 ▶); cell refinement: *SAINT* (Bruker, 2009 ▶); data reduction: *SAINT*; program(s) used to solve structure: *SIR97* (Altomare *et al.*, 1999 ▶); program(s) used to refine structure: *SHELXL97* (Sheldrick, 2008 ▶); molecular graphics: *ORTEP-3 for Windows* (Farrugia, 1997 ▶) and *PLATON* (Spek, 2009 ▶); software used to prepare material for publication: *WinGX* (Farrugia, 1999 ▶) and *PLATON*.

## Supplementary Material

Crystal structure: contains datablock(s) global, I. DOI: 10.1107/S1600536812016479/hg5210sup1.cif

Structure factors: contains datablock(s) I. DOI: 10.1107/S1600536812016479/hg5210Isup2.hkl

Supplementary material file. DOI: 10.1107/S1600536812016479/hg5210Isup3.cml

Additional supplementary materials:  crystallographic information; 3D view; checkCIF report

## Figures and Tables

**Table 1:** Hydrogen-bond geometry (Å, °)

*D*—H⋯*A*	*D*—H	H⋯*A*	*D*⋯*A*	*D*—H⋯*A*
N1—H1*N*⋯S1^i^	0.84 (2)	2.84 (2)	3.669 (2)	168 (2)

Symmetry code: (i) 
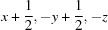
.

**Fig. 1.**
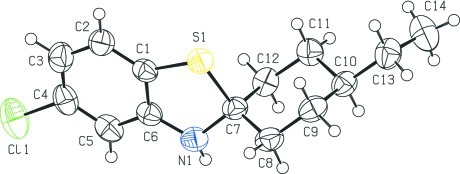
The title compound with the atom numbering scheme. Displacement ellipsoids for non-H atoms are drawn at the 50% probability level.

**Fig. 2.**
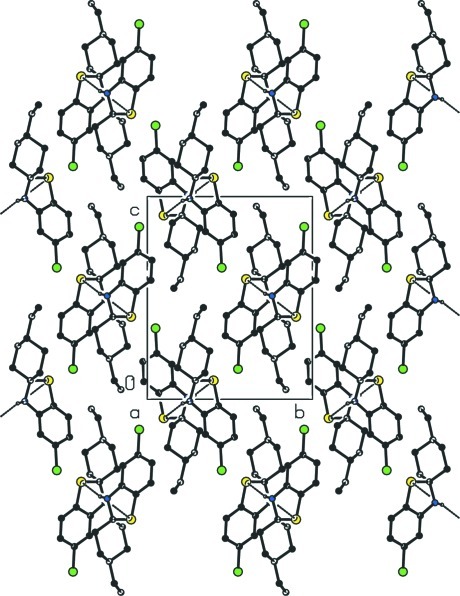
The packing and hydrogen bonding of the title molecule in the unit cell, viewing down a axis. H atoms not involved in hydrogen bonds have been omitted for clarity.

**Fig. 3.**
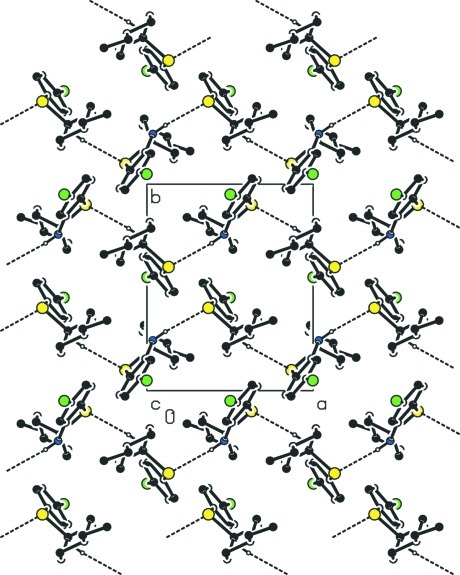
The packing and hydrogen bonding of the title molecule in the unit cell, viewing down c axis. H atoms not involved in hydrogen bonds have been omitted for clarity.

## supplementary crystallographic information

### Comment

Efforts to design, synthesize and screen new molecules that mimic the actions of currently available chemotherapeutics have resulted in numerous promising candidates incorporating the benzothiazole moiety. Benzothiazolines and spirobenzothiazolines, the cyclic products obtained by the condensation of aldehydes and ketones with 2-aminothiophenoles were previously reported to exhibit antitubercular (Palmer *et al.*, 1971), analgesic (Coudert *et al*., 1988) and antioxidant (Karalı *et al.*, 2010) properties. In this context, the title compound was prepared by the condensation of 4-ethylcyclohexanone with 2-amino-4-chlorothiophenol in an attempt to obtain a new molecule with antioxidant action and to establish its definite structure *via* analytical, spectroscopic and crystallographic data.

In the title compound (I), (Fig. 1), the S1/N1/C1/C6/C7 2,3-dihydro-1,3-thiazole ring adopts an envelope conformation with the C7 atom at the flap [puckering parameters (Cremer & Pople, 1975): Q(2) = 0.2817 (18) Å, φ(2) = 144.3 (4) °]. The C7—C12 cyclohexane ring adopts a chair conformation [puckering parameters: Q_T_ = 0.552 (2) Å, θ = 180.0 (2) ° and φ = 337 (7)°]. The bond lengths of (I) are within the expected values (Allen *et al.*, 1987).

In the crystal, molecules are linked by intermolecular N1—H1N···S1 hydrogen bonds (Table 1 and Figs. 2 & 3), forming C(5) motifs (Bernstein *et al.*, 1995) as chains parallel to the *c* axis.

### Experimental

A mixture of 2-amino-4-chlorothiophenol (0.01 mol) and 4-ethylcyclohexanone (0.01 mol) in absolute ethanol (50 ml) was refluxed on a water bath for 8 h. The solvent was evaporated in a crystallizing dish at room temperature and the residue was recrystallized from ethanol. [Yield: 45.5%, m.p.: 381–383 K]. IR (KBr) ν = 3310 (N—H), 2960, 2916, 2893 (C—H), 1585, 1562, 1469, 1448 (C=C) cm^-1^; ^1^H-NMR (DMSO-d_6_, 500 MHz) d = 0.85–0.87 (3*H*, m, 4'-CH_2_—CH_3_-cyc.), 1.00–1.16 (2*H*, m, CH/CH_2_-cyc.), 1.17–1.34 (3*H*, m, 4'-CH_2_—CH_3_ and CH/CH_2_-cyc.), 1.57–1.73 (4*H*, m, CH/CH_2_-cyc.), 2.07–2.13 (2*H*, m, CH/CH_2_-cyc.), 6.41, 6.47–6.50 (2*H*, d, J=2.0 Hz and m, H4 and H6-bt.), 6.90 (1*H*, d, J=8.3 Hz, H7-bt.), 6.74, 6.94 (1*H*, 2 s, NH) p.p.m. (Peaks at d 6.74 and 6.94 disappeared on D_2_O exchange) (cyc.=cyclohexane, bt.=benzothiazole). Analysis calculated for C_14_H_18_ClNS: C 62.79, H 6.77, N 5.23%. Found: C 62.63, H 6.79, N 5.24%.

### Refinement

C-bound H atoms were placed geometrically with the C—H distance of 0.93, 0.96, 0.97 and 0.98 Å, for the aromatic, methyl, methylene and methine H atoms, respectively and refined by using the riding model [*U*_iso_(H) = *xU*_eq_(C), *x* = 1.5 for methyl H and 1.2 for all other carbon-bound H atoms. The nitrogen-bound H atom were located in a difference Fourier map and refined freely with the constraint N—H = 0.86 (2) Å.

### Crystal data

**Table:**

C_14_H_18_ClNS	*F*(000) = 568
*M**_r_* = 267.81	*D*_x_ = 1.292 Mg m^−^^3^
Orthorhombic, *P*2_1_2_1_2_1_	Mo *K*α radiation, λ = 0.71073 Å
Hall symbol: P 2ac 2ab	Cell parameters from 859 reflections
*a* = 8.989 (3) Å	θ = 3.5–20°
*b* = 11.163 (4) Å	µ = 0.41 mm^−^^1^
*c* = 13.722 (4) Å	*T* = 296 K
*V* = 1376.9 (8) Å^3^	Prism, light yellow
*Z* = 4	0.35 × 0.28 × 0.25 mm

### Data collection

**Table:**

Bruker Kappa APEXII CCD diffractometer	3203 independent reflections
Radiation source: fine-focus sealed tube	2724 reflections with *I* > 2σ(*I*)
Graphite monochromator	*R*_int_ = 0.020
ω scans	θ_max_ = 27.9°, θ_min_ = 2.4°
Absorption correction: multi-scan (*SADABS*; Bruker, 2005)	*h* = −11→8
*T*_min_ = 0.872, *T*_max_ = 0.903	*k* = −14→10
7077 measured reflections	*l* = −18→14

### Refinement

**Table:**

Refinement on *F*^2^	Secondary atom site location: difference Fourier map
Least-squares matrix: full	Hydrogen site location: inferred from neighbouring sites
*R*[*F*^2^ > 2σ(*F*^2^)] = 0.038	H atoms treated by a mixture of independent and constrained refinement
*wR*(*F*^2^) = 0.092	*w* = 1/[σ^2^(*F*_o_^2^) + (0.0397*P*)^2^ + 0.2606*P*] where *P* = (*F*_o_^2^ + 2*F*_c_^2^)/3
*S* = 1.03	(Δ/σ)_max_ < 0.001
3203 reflections	Δρ_max_ = 0.32 e Å^−^^3^
159 parameters	Δρ_min_ = −0.35 e Å^−^^3^
1 restraint	Absolute structure: Flack (1983), 1320 Freidel pairs
Primary atom site location: structure-invariant direct methods	Flack parameter: 0.02 (8)

### Special details

**Table:**

Geometry. Bond distances, angles *etc*. have been calculated using the rounded fractional coordinates. All su's are estimated from the variances of the (full) variance-covariance matrix. The cell e.s.d.'s are taken into account in the estimation of distances, angles and torsion angles
Refinement. Refinement on *F*^2^ for ALL reflections except those flagged by the user for potential systematic errors. Weighted *R*-factors *wR* and all goodnesses of fit *S* are based on *F*^2^, conventional *R*-factors *R* are based on *F*, with *F* set to zero for negative *F*^2^. The observed criterion of *F*^2^ > σ(*F*^2^) is used only for calculating -*R*-factor-obs *etc*. and is not relevant to the choice of reflections for refinement. *R*-factors based on *F*^2^ are statistically about twice as large as those based on *F*, and *R*-factors based on ALL data will be even larger.

### Fractional atomic coordinates and isotropic or equivalent isotropic displacement parameters (Å^2^)

**Table:**

	*x*	*y*	*z*	*U*_iso_*/*U*_eq_	
Cl1	0.49813 (9)	0.45066 (8)	−0.35059 (4)	0.0819 (3)	
S1	0.37029 (6)	0.40096 (5)	0.09263 (4)	0.0481 (2)	
N1	0.5350 (2)	0.25670 (18)	−0.01023 (14)	0.0529 (6)	
C1	0.3980 (2)	0.43112 (18)	−0.03208 (14)	0.0430 (7)	
C2	0.3365 (2)	0.5213 (2)	−0.08691 (16)	0.0540 (7)	
C3	0.3676 (3)	0.5274 (2)	−0.18623 (17)	0.0597 (8)	
C4	0.4593 (3)	0.4430 (2)	−0.22640 (16)	0.0547 (8)	
C5	0.5230 (3)	0.3517 (2)	−0.17270 (16)	0.0527 (7)	
C6	0.4912 (2)	0.34542 (18)	−0.07400 (14)	0.0437 (6)	
C7	0.5242 (2)	0.29047 (17)	0.09277 (15)	0.0437 (6)	
C8	0.4828 (3)	0.18328 (18)	0.15561 (16)	0.0534 (7)	
C9	0.4758 (3)	0.21449 (19)	0.26303 (16)	0.0541 (7)	
C10	0.6208 (3)	0.26917 (19)	0.30042 (15)	0.0470 (6)	
C11	0.6605 (2)	0.3776 (2)	0.23844 (15)	0.0506 (7)	
C12	0.6673 (2)	0.3484 (2)	0.12979 (15)	0.0495 (7)	
C13	0.6130 (3)	0.2992 (2)	0.40837 (16)	0.0602 (8)	
C14	0.7593 (3)	0.3396 (3)	0.4523 (2)	0.0791 (10)	
H1N	0.611 (2)	0.214 (2)	−0.0203 (19)	0.078 (9)*	
H2	0.27450	0.57770	−0.05800	0.0650*	
H3	0.32680	0.58780	−0.22450	0.0720*	
H5	0.58560	0.29600	−0.20200	0.0630*	
H8A	0.38670	0.15290	0.13500	0.0640*	
H8B	0.55560	0.12030	0.14590	0.0640*	
H9A	0.45400	0.14260	0.30000	0.0650*	
H9B	0.39520	0.27080	0.27370	0.0650*	
H10	0.69960	0.20940	0.29160	0.0560*	
H11A	0.75630	0.40850	0.25920	0.0610*	
H11B	0.58700	0.43990	0.24900	0.0610*	
H12A	0.68540	0.42150	0.09350	0.0590*	
H12B	0.75000	0.29450	0.11790	0.0590*	
H13A	0.53990	0.36200	0.41780	0.0720*	
H13B	0.57860	0.22900	0.44340	0.0720*	
H14A	0.83630	0.28400	0.43480	0.1190*	
H14B	0.75030	0.34280	0.52190	0.1190*	
H14C	0.78410	0.41770	0.42790	0.1190*	

### Atomic displacement parameters (Å^2^)

**Table:**

	*U*^11^	*U*^22^	*U*^33^	*U*^12^	*U*^13^	*U*^23^
Cl1	0.0729 (4)	0.1232 (6)	0.0495 (3)	−0.0171 (5)	0.0012 (3)	0.0056 (3)
S1	0.0423 (3)	0.0527 (3)	0.0492 (3)	0.0097 (3)	0.0031 (2)	−0.0056 (2)
N1	0.0541 (12)	0.0517 (11)	0.0528 (10)	0.0153 (10)	0.0027 (9)	−0.0101 (8)
C1	0.0378 (11)	0.0448 (12)	0.0464 (11)	−0.0016 (9)	−0.0028 (8)	−0.0087 (8)
C2	0.0495 (12)	0.0538 (13)	0.0586 (13)	0.0064 (10)	−0.0104 (11)	−0.0055 (11)
C3	0.0562 (13)	0.0646 (15)	0.0582 (13)	−0.0029 (13)	−0.0128 (12)	0.0067 (11)
C4	0.0476 (13)	0.0704 (15)	0.0462 (11)	−0.0142 (12)	−0.0063 (9)	−0.0003 (11)
C5	0.0423 (11)	0.0642 (14)	0.0517 (12)	−0.0016 (11)	0.0036 (10)	−0.0130 (11)
C6	0.0356 (10)	0.0465 (11)	0.0489 (11)	−0.0031 (9)	−0.0018 (9)	−0.0076 (9)
C7	0.0421 (11)	0.0398 (10)	0.0492 (11)	0.0056 (9)	0.0007 (10)	−0.0034 (9)
C8	0.0575 (13)	0.0375 (11)	0.0653 (14)	−0.0031 (10)	−0.0026 (12)	−0.0005 (9)
C9	0.0580 (14)	0.0426 (11)	0.0618 (13)	−0.0060 (11)	0.0061 (11)	0.0093 (10)
C10	0.0444 (11)	0.0463 (11)	0.0502 (11)	0.0066 (10)	0.0034 (10)	0.0035 (9)
C11	0.0450 (12)	0.0530 (13)	0.0537 (12)	−0.0103 (10)	−0.0018 (9)	0.0023 (10)
C12	0.0386 (11)	0.0559 (13)	0.0540 (12)	−0.0016 (10)	0.0030 (9)	0.0083 (10)
C13	0.0590 (14)	0.0690 (15)	0.0525 (13)	0.0078 (13)	0.0031 (12)	0.0082 (12)
C14	0.0742 (18)	0.104 (2)	0.0591 (15)	0.0009 (18)	−0.0082 (14)	−0.0086 (16)

### Geometric parameters (Å, º)

**Table:**

Cl1—C4	1.742 (2)	C13—C14	1.515 (4)
S1—C1	1.762 (2)	C2—H2	0.9300
S1—C7	1.854 (2)	C3—H3	0.9300
N1—C6	1.379 (3)	C5—H5	0.9300
N1—C7	1.466 (3)	C8—H8A	0.9700
N1—H1N	0.84 (2)	C8—H8B	0.9700
C1—C6	1.396 (3)	C9—H9A	0.9700
C1—C2	1.373 (3)	C9—H9B	0.9700
C2—C3	1.393 (3)	C10—H10	0.9800
C3—C4	1.368 (3)	C11—H11A	0.9700
C4—C5	1.382 (3)	C11—H11B	0.9700
C5—C6	1.386 (3)	C12—H12A	0.9700
C7—C8	1.521 (3)	C12—H12B	0.9700
C7—C12	1.527 (3)	C13—H13A	0.9700
C8—C9	1.516 (3)	C13—H13B	0.9700
C9—C10	1.528 (4)	C14—H14A	0.9600
C10—C11	1.522 (3)	C14—H14B	0.9600
C10—C13	1.520 (3)	C14—H14C	0.9600
C11—C12	1.527 (3)		
			
C1—S1—C7	91.30 (9)	C6—C5—H5	121.00
C6—N1—C7	114.09 (17)	C7—C8—H8A	109.00
C6—N1—H1N	122.2 (17)	C7—C8—H8B	109.00
C7—N1—H1N	110.9 (18)	C9—C8—H8A	109.00
S1—C1—C2	127.99 (16)	C9—C8—H8B	109.00
S1—C1—C6	110.75 (14)	H8A—C8—H8B	108.00
C2—C1—C6	121.23 (18)	C8—C9—H9A	109.00
C1—C2—C3	119.4 (2)	C8—C9—H9B	109.00
C2—C3—C4	118.8 (2)	C10—C9—H9A	109.00
Cl1—C4—C5	118.36 (18)	C10—C9—H9B	109.00
C3—C4—C5	122.9 (2)	H9A—C9—H9B	108.00
Cl1—C4—C3	118.77 (18)	C9—C10—H10	108.00
C4—C5—C6	118.2 (2)	C11—C10—H10	108.00
N1—C6—C5	126.69 (19)	C13—C10—H10	108.00
N1—C6—C1	113.71 (17)	C10—C11—H11A	109.00
C1—C6—C5	119.46 (19)	C10—C11—H11B	109.00
S1—C7—C12	110.33 (14)	C12—C11—H11A	109.00
N1—C7—C8	111.13 (16)	C12—C11—H11B	109.00
S1—C7—C8	109.97 (14)	H11A—C11—H11B	108.00
C8—C7—C12	110.53 (17)	C7—C12—H12A	109.00
N1—C7—C12	111.96 (16)	C7—C12—H12B	109.00
S1—C7—N1	102.68 (13)	C11—C12—H12A	109.00
C7—C8—C9	112.37 (17)	C11—C12—H12B	109.00
C8—C9—C10	112.5 (2)	H12A—C12—H12B	108.00
C9—C10—C13	112.1 (2)	C10—C13—H13A	109.00
C11—C10—C13	112.33 (18)	C10—C13—H13B	109.00
C9—C10—C11	109.28 (18)	C14—C13—H13A	109.00
C10—C11—C12	112.66 (18)	C14—C13—H13B	109.00
C7—C12—C11	112.43 (16)	H13A—C13—H13B	108.00
C10—C13—C14	114.4 (2)	C13—C14—H14A	109.00
C1—C2—H2	120.00	C13—C14—H14B	110.00
C3—C2—H2	120.00	C13—C14—H14C	109.00
C2—C3—H3	121.00	H14A—C14—H14B	110.00
C4—C3—H3	121.00	H14A—C14—H14C	109.00
C4—C5—H5	121.00	H14B—C14—H14C	109.00
			
C7—S1—C1—C2	−169.63 (19)	Cl1—C4—C5—C6	−179.63 (18)
C7—S1—C1—C6	12.78 (15)	C3—C4—C5—C6	0.5 (4)
C1—S1—C7—N1	−22.42 (13)	C4—C5—C6—N1	175.0 (2)
C1—S1—C7—C8	−140.76 (15)	C4—C5—C6—C1	−0.6 (3)
C1—S1—C7—C12	97.06 (15)	S1—C7—C8—C9	−68.9 (2)
C6—N1—C7—S1	28.68 (19)	N1—C7—C8—C9	178.09 (19)
C6—N1—C7—C8	146.21 (18)	C12—C7—C8—C9	53.2 (3)
C6—N1—C7—C12	−89.7 (2)	S1—C7—C12—C11	69.5 (2)
C7—N1—C6—C1	−21.5 (2)	N1—C7—C12—C11	−176.80 (17)
C7—N1—C6—C5	162.7 (2)	C8—C7—C12—C11	−52.3 (2)
S1—C1—C2—C3	−177.34 (17)	C7—C8—C9—C10	−56.0 (3)
C6—C1—C2—C3	0.0 (3)	C8—C9—C10—C11	55.3 (2)
S1—C1—C6—N1	2.0 (2)	C8—C9—C10—C13	−179.54 (18)
S1—C1—C6—C5	178.12 (17)	C9—C10—C11—C12	−54.5 (2)
C2—C1—C6—N1	−175.77 (18)	C13—C10—C11—C12	−179.53 (19)
C2—C1—C6—C5	0.3 (3)	C9—C10—C13—C14	172.9 (2)
C1—C2—C3—C4	−0.1 (3)	C11—C10—C13—C14	−63.6 (3)
C2—C3—C4—C5	−0.1 (4)	C10—C11—C12—C7	54.5 (2)
C2—C3—C4—Cl1	179.98 (18)		

### Hydrogen-bond geometry (Å, º)

**Table:**

*D*—H···*A*	*D*—H	H···*A*	*D*···*A*	*D*—H···*A*
N1—H1*N*···S1^i^	0.84 (2)	2.84 (2)	3.669 (2)	168 (2)

Symmetry code: (i) *x*+1/2, −*y*+1/2, −*z*.
